# Evaluation of *MMP1* and *MMP3* gene polymorphisms in exfoliation syndrome and exfoliation glaucoma

**Published:** 2009-12-25

**Authors:** Evangelia E. Tsironi, Maria Pefkianaki, Aspasia Tsezou, Maria G. Kotoula, Efthimios Dardiotis, Pavlina Almpanidou, Afroditi A. Papathanasiou, Paraskevi Rodopoulou, Dimitrios Z. Chatzoulis, Georgios M. Hadjigeorgiou

**Affiliations:** 1 Department of Ophthalmology, School of Medicine, University of Thessaly, Larissa, Greece; 2 Department of Biology, School of Medicine, University of Thessaly, Larissa, Greece; 3 Institute for Biomedical Research & Technology (BIOMED), Centre for Research and Technology-Thessaly (CERETETH), Larissa, Greece; 4 Department of Biomathematics, School of Medicine, University of Thessaly, Larissa, Greece; 5 Department of Neurology, School of Medicine, University of Thessaly, Larissa, Greece

## Abstract

**Purpose:**

To investigate possible genetic associations of matrix metalloproteinase-1 (*MMP1*) and *MMP3* gene polymorphisms with exfoliation syndrome (XFS) with (XFS/+G) and without (XFS/-G) glaucoma in a cohort of Greek patients.

**Methods:**

A total of 182 unrelated Greek patients with XFS, including 92 patients with XFS/+G, and 214 unrelated age- and gender-matched controls were enrolled in the study. *MMP1* -1607 1G/2G (rs1799750) and *MMP3* -1171 5A/6A (rs3025058) polymorphisms were determined using standard PCR/restriction fragment length polymorphism methods. Differences in allele and genotype distributions were analyzed using logistic regression.

**Results:**

The distribution of genotypes and alleles in *MMP1* and *MMP3* polymorphisms was not significantly different between cases with exfoliation syndrome, with or without glaucoma, and controls. However, the allele contrast for the *MMP1* variant showed a trend for a significant association with XFS/-G (Odds Ratio=1.47 [1.03–2.10]), since after correction for multiple comparisons, this association was no longer statistically significant.

**Conclusions:**

Our study provided some evidence of a possible role of the *MMP1* variant in the development of exfoliation syndrome in Greek patients.

## Introduction

Exfoliation syndrome (XFS) is an age-related generalized disorder of the extracellular matrix associated with excessive synthesis and progressive deposition of a fibrillar material in the ocular tissues as well as in the skin and connective tissue of various visceral organs [1]. It is characterized by small whitish deposits of fibrillar-granular material in the anterior segment of the eye. Its ocular manifestations involve all the structures of the anterior segment as well as the conjunctiva and the orbital tissues [2]. An increased number of associations of XFS with specific systemic disorders [1] has been reported.

Raised intraocular pressure develops in a significant number of patients with exfoliation syndrome. Exfoliation glaucoma (XFS/+G) is a common sight-threatening disease that develops as a consequence of exfoliation syndrome. There are important differences in the clinical appearance, course, and prognosis of exfoliative glaucoma versus primary open-angle glaucoma. On clinical, biochemical, and cellular levels, XFS/+G is a distinct entity with an intriguing mechanism of development and numerous systemic manifestations that require further elucidation [3].

Extracellular matrix turnover is mediated by matrix metalloproteinases (MMPs), a large family of endopeptidases with variable substrate spectra [4]. The activity of these enzymes is regulated in part by specific endogenous inhibitors, the tissue inhibitors of metalloproteinases (TIMPs). Complex changes in the local MMP–TIMP are possible candidates to be involved in the abnormal extracellular matrix metabolism characteristic of XFS and XFS/+G. Increased biosynthesis of MMPs in eyes with XFS has been reported, and this may serve to degrade the abnormal fibrillar matrix components in the anterior segment tissues [5]. However, the increased levels of MMPs do not seem to be able to overcome the overproduction and accumulation of exfoliative material [5]. Growing evidence indicates that spontaneous sequence variations in the promoters of MMPs may influence critical steps in the binding of transcription factors or overall transcriptional efficiency, which results in differential expression of MMPs in different individuals [6].

An insertion (2G)/deletion (1G) polymorphism (rs1799750) of *MMP1* (OMIM 120353, chr.11q22-q23) within the promoter region at position -1607 bp influences the transcription of this *MMP1* gene; the 2G promoter processes greater transcriptional activity than the 1G promoter [7,8].

There is also a single adenine insertion (6A) or deletion (5A) polymorphism (rs3025058) at position -1171 bp of the *MMP3* (OMIM 185250, chr.11q23) promoter that influences *MMP3* expression by changing the affinity of the repressor binding site, with the 6A allele sequence having a stronger recognition for the repressor binding site. This leads to enhanced transcriptional levels of *MMP3* in the presence of the 5A allele [6,9]. In addition, both *MMP1* and *MMP3* are known to be adjacently localized in chromosome 11q22.3, and these two loci are in linkage disequilibrium and considered to act in cooperation [10].

Our study was designed to investigate whether the insertion/deletion polymorphisms of the *MMP1* and *MMP3* promoters have any correlation with the development of XFS, and XFS/+G in particular. To our knowledge, this is the first study that has evaluated the association between *MMP1* and *MMP3* polymorphisms and XFS and XFS/+G.

## Methods

### Patients and control subjects

This association study included 182 patients with unilateral or bilateral XFS, 92 of which had unilateral or bilateral exfoliation glaucoma (XFS/+G), 90 without glaucoma (XFS/-G), and 214 age- and gender-matched control subjects. All subjects were recruited from the Ophthalmologic Department of the University Hospital of Larissa (a tertiary referral center in central Greece that covers a population of almost one million inhabitants). The study was approved by the local ethics committee and carried out according to the Declaration of Helsinki. All subjects were of Greek nationality from the same geographic region and gave informed consent before entering the study. The age and sex distribution of the patients and controls are shown in Table 1.

**Table 1:** Clinical characteristics of study populations.

**Gender/Age**	**XFS Total (n=182)**	**XFS/+G (n=92)**	**XFS/-G (n=90)**	**Controls (n=214)**
Female (%)	111 (60.9)	58 (63.1)	53 (58.9)	122 (57.0)
Age±SD (years)	72.0±7.1	71.3±7.7	72.9±6.1	71.8±7.8

In the table, XFS refers to exfoliation syndrome, XFS/+G refers to exfoliation syndrome with glaucoma, and XFS/-G refers to exfoliation syndrome without glaucoma.

All subjects underwent detailed ophthalmic examination by the same investigator (P.M.). Patients or controls with any type of malignancy, autoimmune disease, ocular disease, history of cardiovascular disease, neurologic disease, obstructive pulmonary disease, diabetes mellitus, hepatitis, or allergies were not included in the study because these conditions have been associated with abnormal expression of MMPs or their inhibitors [11].

In a detailed ophthalmologic examination, the anterior and posterior segments of all cases were evaluated, and intraocular pressures (IOPs) were measured with Goldmann applanation tonometry, manufactured by Haag-Streit (Koeniz-Berne Switzerland). The corneal endothelium, iris, iris margins, and the anterior lens surface were evaluated for exfoliative material before and after dilation. To evaluate the angle for exfoliative material and increased pigmentation, gonioprisms were used.

Patients were classified as having unilateral or bilateral XFS by the presence of characteristic fibrillar-exfoliate material on the anterior lens capsule or pupillary margin and by an open anterior chamber angle. Unilateral or bilateral XFS/+G was manifested if patients had XFS as well as an IOP greater than 22 mmHg in each eye, typical glaucomatous optic nerve changes (notching, thinner neuroretinal rim, and increased cup-to-disc ratio), and the presence of visual field defects, as determined by a Humphrey visual field analyzer, (full threshold program 24-2; Carl Zeiss Inc., Dublin, CA), consistent with the glaucomatous cupping in at least one eye. The mean IOP at diagnosis was 17±4 mmHg in the patients with XFS and 35.0±13.0 mm Hg in the patients with XFS/+G.

Control subjects had no evidence of exfoliative material at the anterior lens capsule or pupillary margin. In addition the IOP in control subjects was less than 22 mm Hg; control subjects had normal visual fields, an open anterior chamber angle, no evidence of glaucomatous changes in the optic disc, and no history of glaucoma or ocular hypertension in first-degree relatives.

### *MMP1* and *MMP3* polymorphisms

Genomic DNA was extracted from peripheral blood leukocytes using a commercially available DNA extraction kit (Qiagen, Valencia, CA), according to the manufacturer’s instructions. Based on the sequences of *MMP1 *and* MMP3* available from GenBank and using Primer3 software, appropriate primers were designed for *MMP1* (1G/2G, -1607) and *MMP3* (5A/6A, -1171) polymorphisms (Table 2). Thermal cycling conditions were as follows: 15 min at 95 ^o^C, followed by 35 cycles of 30 s at 95 ^o^C, 30 s at 56 ^o^C (*MMP1*) or 30 s at 65 ^o^C (*MMP3*), 30 s at 72 ^o^C, and with a final extension at 72 ^o^C for 5 min. PCR products were digested for 16 h at 37 ^o^C in a 24-μl reaction containing 2 μl AluI and 2 μl ΤthIII restriction enzymes for the determination of *MMP1* and *MMP3* genotypes, respectively. The digested products were subjected to gel electrophoresis and were visualized by ethidium bromide staining. The sizes of PCR products for *MMP1* (1G/2G, -1607) and *MMP3* (5A/6A, -1171) were 269 and 129 bp, respectively.

**Table 2:** Primer sequences for *MMP1* (1G/2G, -1607) and *MMP3* (5A/6A, -1171) polymorphisms.

**Gene**	**Polymorphism**	**Forward primer (5’-3’)**	**Reverse primer 5’-3’**
*MMP1*	1G/2G (rs1799750)	TGACTTTTAAAACATAGTCTATGTTCA	TCTTGGATTGATTTGAGATAAGTCATAGC
*MMP3*	5A/6A (rs3025058)	GGTTCTCCATTCCTTTGATGGGGGGAAAGA	CTTCCTGGAATTCACATCACTGCCACCACT

### Statistical analysis

The Hardy–Weinberg (H-W) equilibrium [12] was assessed by means of an exact H-W test, implemented in the GENEPOP version 3.4 program. Power was calculated using the CaTS Power Calculator for Genetic Studies (Center for Statistical Genetics, University of Michigan, Ann Arbor, MI). Continuous variables were compared (XFS total, XFS/+G, or XFS/-G versus controls) using the nonparametric Mann–Whitney *U* test, and categorical variables were compared using the χ^2^ test. Differences in allele and genotype distributions were analyzed using logistic regression. The odds ratio (OR) and 95% confidence interval (CI) were used as a measure of strength of association between polymorphisms and disease. In the logistic regression, the disease status (patients versus controls) was used as a dichotomous outcome, while allele or genotype was used as a predictor. We additionally controlled for potential confounders, such as age and gender. Later analyses were carried out using SPSS ver. 12.0 (SPSS, Chicago, IL). Control for multiple comparisons was performed using Bonferronis correction for two polymorphisms. The level of significance (p) was equal to 0.025 (0.05/2=0.025).

## Results

Patients and controls were in Hardy–Weinberg equilibrium (p>0.05 for all groups). Differences in mean age and sex distribution between all patients and controls were not significant (p>0.05). Distribution of genotypes and alleles are shown in Table 3. In both polymorphisms (*MMP1* and *MMP3*), the genotype distribution of the XFS total group was not significantly different from controls (p=0.19 and p=0.99). Also, the genotype distributions of XFS/+G and XFS/-G groups were not significantly different from controls (*MMP1* p=0.10 and 0.70, respectively, and *MMP3* p=0.94 and 0.48, respectively).

**Table 3:** Genotype and allele distribution frequencies of the *MMP1* -1607 polymorphism and the *MMP3* -1171 polymorphism.

**Genotypes/Alleles**	**XFS Total (n=182)**	**p-value**	**XFS/+G (n=92)**	**p-value**	**XFS/-G (n=90)**	**p-value**	**Controls (n=214)**
***MMP-1***							
2G/2G (%)	24 (13.2)		11 (11.9)		13 (14.4)		39 (18.2)
2G/1G (%)	89 (48.9)		42 (45.7)		47 (52.3)		110 (51.4)
1G/1G (%)	69 (37.9)	0.19	39 (42.4)	0.10	30 (33.3)	0.70	65 (30.4)
1G (%)	227 (62.4)		120 (65.2)		107 (59.5)		240 (56.1)
2G (%)	137 (37.6)	0.07	64 (34.8)	0.04	73 (40.5)	0.44	188 (43.9)
***MMP-3***							
6A/6A (%)	42 (23.1)		25 (27.2)		17 (18.9)		54 (25.2)
6A/5A (%)	87 (47.8)		42 (45.6)		45 (50.0)		101 (47.2)
5A/5A (%)	53 (29.1)	0.99	25 (27.2)	0.94	28 (31.1)	0.48	59 (27.6)
5A (%)	193 (53.0)		92 (50.0)		101 (56.1)		219 (51.2)
6A (%)	171 (47.0)	0.99	92 (50.0)	0.79	79 (43.9)	0.29	209 (48.8)

Genotype and allele distribution frequencies of the *MMP1* -1607 polymorphism and the *MMP3* -1171 polymorphism in the exfoliation syndrome (XFS Total), exfoliation syndrome with glaucoma (XFS/+G), exfoliation glaucoma without glaucoma (XFS/-G and control groups. The p-values for comparing each diseased group with the controls are also shown.

The results of the influence of genotypes on disease susceptibility based on carrier status of risk allele and considering the allele contrast and the dominant and recessive models are shown in Table 4. The allele contrast for the *MMP1* variant showed a trend for a significant association for XFS/-G (1.47 [1.03–2.10]; p=0.04). This association is considered nonsignificant after correction for multiple comparisons (level of significant <0.025). However, the dominant and recessive models showed that *MMP1* was not associated with XFS total (OR=1.51 [0.87–2.46] and OR=0.73 [0.46–1.13], respectively). The *MMP3* variant was not associated with XFS total for the allele contrast and the dominant and recessive models (OR=0.90 [0.68–1.19]; OR=1.09 [0.68n–1.65]; and OR=1.03 [0.66–1.54], respectively). The subgroups XFS/+G and XFS/-G produced similar results to the XFS total for both variants.

**Table 4:** Influence of genotypes on disease susceptibility based on carrier status of risk allele considering dominant or recessive model.

**Model**	***MMP-1* (-1607 1G/2G)**				***MMP-3* (-1171 5A/6A)**			
**Allele contrast**	**1G**	**2G**	OR (95%CI)*	**p**	**6A**	**5A**	OR (95%CI)*	**p**
Controls	240	188	Reference		209	219	Reference	
Patients-XFS/+G	120	64	1.47 (1.03-2.10)	0.04	92	92	0.95 (0.68-1.35)	0.79
Patients-XFS/-G	107	73	1.15 (0.81-1.64)	0.44	79	101	0.75 (0.53-1.06)	0.29
Patients-XFS total	227	137	1.30 (0.98-1.73)	0.07	171	193	0.90 (0.68-1.19)	0.99
**Dominant model**	**1G/-**	**-/-**	OR (95%CI)*	**p**	**5A/-**	**-/-**	OR(95%CI)*	**p**
Controls	175	39	Reference		160	54	Reference	
Patients-XFS/+G	81	11	1.61 (0.78-3.35)	0.18	67	25	0.89 (0.50-1.53)	0.68
Patients-XFS/-G	77	13	1.33 (0.68-2.65)	0.46	73	17	1.42 (0.74-2.61)	0.25
Patients-XFS total	158	24	1.51 (0.87-2.46)	0.20	140	42	1.09 (0.68-1.65)	0.91
**Recessive model**	**1G/1G**	**2G/-**	OR (95%CI)*	** p**	**5A/5A**	**6A/-**	OR (95%CI)*	** p**
Controls	65	149	Reference		59	155	Reference	
Patients-XFS/+G	39	53	0.62 (0.43-0.98)	0.05	25	67	1.04 (0.58-1.73)	0.93
Patients-XFS/-G	30	60	0.90 (0.50-1.48)	0.65	28	62	0.86 (0.49-1.38)	0.57
Patients-XFS total	69	113	0.73 (0.46-1.13)	0.13	53	129	1.03 (0.66-1.54)	0.97

The asterisk indicates that OR was adjusted for age and gender.

## Discussion

XFS is a complex disorder with both genetic and nongenetic factors involved in its development and progression. Among nongenetic factors, ultraviolet light, autoimmunity, slow virus infection, and trauma have been suggested as contributing factors to XFS [13].

Exfoliation syndrome is often clustered in families, so it has been proposed that genetic factors play an important role in its cause [14]. In an Icelandic twin study, five of eight monozygotic twins with exfoliation were concordant for XFS [15], as was the case in another small case study of monozygotic twins [14]. Segregation analyses conducted on the Finnish population clearly showed heredity to be a primary contributor to XFS susceptibility [14], and recently the susceptible loci were proposed to be 18q12.1–21.33, 2q, 17p, and 19q.9 [16].

In addition, genetic association studies have been conducted to investigate the association between genetic polymorphisms and XFS with or without glaucoma. Recent studies showed that lysil oxidase-like 1 (*LOX1*) gene polymorphisms are highly associated with exfoliation phenotype [17–20]. Replication studies in other populations have confirmed genetic susceptibility of *LOXL1* polymorphisms to XFS [17,21,22], demonstrating that *LOXL1* is a major gene associated with XFS. Polymorphisms in glutathione S-transferase (*GST*) genes (*GSTM1*, *GSTT1*, *GSTP1*) [23] and the tumor necrosis factor-α gene *(TNFα)* -308 G/A have also been tested in XFS [24,25].

MMPs and their inhibitors are shown to play an important role in the development of an abnormal extracellular matrix characteristic of XFS/XFS/+G [2,26]. Increased biosynthesis of MMPs has been found in XFS, which may limit the progression of XFS by degrading the abnormal fibrillar matrix components in the anterior segment tissues; however, it was also suggested the MMPs may have a role in promoting exfoliation [5]. *MMP1* (1G/2G, -1607) and *MMP3* (5A/6A, -1171) functional polymorphisms have been studied in various diseases and in several populations [6]. They have been associated, among others, with increased risk for colorectal cancer [10], carotid artery disease [27], acute myocardial infarction [28], and idiopathic dilated cardiomyopathy [29].

In the present study, we determined the genotypes of two functional polymorphisms, *MMP1* (1G/2G, -1607) and *MMP3* (5A/6A, -1171), in healthy individuals and in patients with XFS and XFS/+G, and for the allele contrast we found a trend for association only between the *MMP1* variant and XFS without glaucoma.

We should underscore that the sample size in our study was small, and thus the power of detecting significant results was limited (almost 20%). However, candidate-gene association studies have the tendency to lack the power to detect a statistically significant association. For example, in order to achieve a power >80% to identify a modest genetic effect (odds ratio 1.2) of a polymorphism present in 10% of individuals, a sample size of 10,000 subjects or more would be needed [30,31]. Therefore, the sample sizes required to predict association have to be far beyond what is currently available, and no single institution or entity alone would be able to provide a reasonable number of patients. However, a future meta-analysis of multiple studies clearly has a role in offering an analysis with the potential for higher power [32]. Future collaborative studies may allow the pooling of data, providing more power to detect significant associations. Furthermore, consortia performing gene-candidate or genome-wide association studies will be able to replicate the validity of the present findings [33].

Nevertheless, the present study fulfills the minimum requirements for the association study to be informative [32]. These include population-based matched controls, good scientific rationale, presence of H–W equilibrium in genotypes, and a similar ethnic background. In parallel, our study has all the limitations that are applicable to genetic association studies [34]. Because our hospital is the only tertiary institution covering a rural and urban region with nearly 1 million inhabitants, patients included in this study are population-based XFS patients. For this reason, more severe patients are usually referred to our hospital, and we cannot exclude the possibility that a selection bias may have occurred.

In summary, our study provided some evidence of a possible significant role of the *MMP1* variant in the development of exfoliation syndrome in Greek patients. However, as we cannot exclude the possibility that our findings were due to random events or type 1 error, further studies using large cohorts of patients are needed to explore the possible genetic involvement of the above polymorphisms in the exfoliation syndrome.
